# Organizing pneumonia in ALK+ lung adenocarcinoma treated with ceritinib: A case report and literature review

**DOI:** 10.1097/MD.0000000000026449

**Published:** 2021-07-02

**Authors:** Yonghui Wu, Huiguo Chen, Jiexia Guan, Kai Zhang, Weibin Wu, Xiaojun Li, Jian Zhang

**Affiliations:** a Department of Cardiothoracic Surgery; b Department of Pathology, the Third Affiliated Hospital of Sun Yat-Sen University, Guangzhou, China.

**Keywords:** adverse events, ALK rearrangement, case report, ceritinib, organizing pneumonia

## Abstract

**Rationale:**

Anaplastic lymphoma kinase (ALK) inhibitors have been approved for patients with ALK-rearrangement lung cancer. The effect is superior to the standard first-line therapy of pemetrexed plus platinum-based chemotherapy. However, ALK inhibitors are associated with rare and sometimes fatal adverse events. Organizing pneumonitis (OP) is a rare and serious adverse event usually caused by ceritinib, and it is easily misdiagnosed as infectious pneumonia, metastasis, or cancer progression.

**Patient concerns:**

A 56-year-old female presented with chest tightness and dyspnea for more than 10 days. She was previously healthy with no significant medical history. Workup including chest computed tomography (CT), pathological examination of a biopsy specimen, and next-generation sequencing was consistent with a diagnosis of IVA ALK-rearrangement lung adenocarcinoma. She was treated with pemetrexed plus platinum-based chemotherapy and crizotinib concurrently, followed by maintenance therapy with crizotinib alone and she had an almost complete response. However, about 26 months after beginning treatment she developed multiple brain metastases. Crizotinib was discontinued and she was begun on ceritinib. After about 3 months the brain metastases had almost complete response. After 5 months of ceritinib, however, multiple patchy lesions appeared in the bilateral upper lungs.

**Diagnoses:**

Treatment with antibiotics had no effect and blood and sputum cultures are negative. A CT-guided biopsy of the upper lung was performed, and pathological hematoxylin-eosin staining and immunohistochemical studies were consistent with OP.

**Interventions:**

Ceritinib was discontinued, she was begun on prednisone 0.5 mg/kg orally every day, and regular follow-up is necessary.

**Outcomes:**

CT of the chest 2 and 4 weeks after beginning prednisone showed the lung lesions to be gradually resolving, and she was continued on prednisone for 2 months and gradually reduced the dose of prednisone every 2 weeks. No related adverse events were occurred in patient.

**Lessons:**

OP must be differentiated from infectious pneumonia, metastasis, or cancer progression. The mechanism of OP is still unknown and needs further research. Biopsy plays a role in making a diagnosis of OP. In our patient, discontinuing ceritinib and treating her with prednisone resulted in a good outcome.

## 1 Introduction

Lung cancer is associated with the highest morbidity and mortality rates of any malignancy. Approximately 80% to 85% of lung cancers are non-small cell lung cancer (NSCLC). In recent decades, the development of targeted therapies has prolonged progression-free survival (PFS) and overall survival (OS) of patients with lung cancer and molecular mutations, especially adenocarcinoma.^[1]^

The echinoderm microtubule-associated protein-like 4-anaplastic lymphoma kinase (ALK) fusion gene is presented in 3% to 7% of NSCLC, and is an important molecular mutation.^[2–4]^ Crizotinib is a first-generation ALK inhibitor, and was first approved for the treatment of patients with ALK-rearrangement (ALK+). Ceritinib, alectinib, brigatinib, and ensartinib are second-generation ALK inhibitors, and lorlatinib is a third-generation ALK inhibitor, and all have been used to treat patients with ALK+ lung cancer.^[3]^ ALK inhibitors have been shown to improve PFS and OS compared with standard first-line therapy of pemetrexed plus platinum-based chemotherapy.^[5]^ However, eventual drug resistance is common with crizotinib, while second- and third-generation ALK inhibitors can also improve PFS and OS of patient.

Ceritinib was approved in April, 2014, and is used to treat patients with ALK+ advanced NSCLC who have progressed on crizotinib, or who cannot tolerate crizotinib.^[6]^ Ceritinib exhibits marked antitumor activity against ALK+ NSCLC, as well as brain metastases; however, it is associated with a number of adverse events including diarrhea, nausea, prolonged QT interval, and fatal pneumonitis.^[7]^ Organizing pneumonitis (OP) is a very rare adverse event, and usual is commonly misdiagnosed infectious pneumonia, metastases, or cancer progression.^[8–10]^ A diagnosis of OP should be suspected when there is no response to multiple antibiotics, and blood and sputum cultures are negative. It affects the patient prognosis, and its treatment is different than pneumonia and cancer progression; thus, a prompt and accurate diagnosis is very important.

There are currently only a few reports of OP associated with ceritinib. Herein, we report the case of a female with lung adenocarcinoma who developed OP while being treated with ceritinib.

## 2 Case presentation

**Figure 1:**
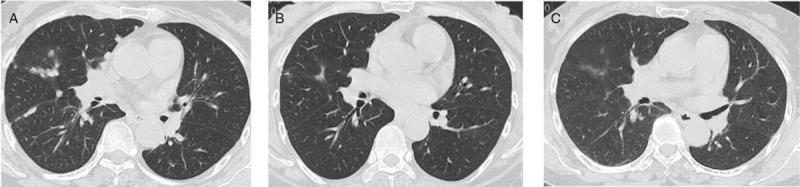
A, The first chest CT scan. B, Two cycles of chemotherapy and crizotinib. C, Six cycles of chemotherapy and crizotinib. CT = computer tomography.

A 56-year-old female presented to our hospital on April 15, 2018, with a complaint of chest tightness and dyspnea for more than 10 days. Chest computed tomography (CT) demonstrated a large of right pleural effusion and multiple nodules consistent with tumors in the right lung lobes. She was a nonsmoker, had no family history of malignancy, and was otherwise healthy with no significant medical history. Her blood carcinoembryonic antigen (CEA) level was 603.44 μg/L, and the level in the pleural effusion was 1162.98 μg/L. Pathological examination of the pleural effusion was consistent with adenocarcinoma. Next-generation sequencing (NGS) revealed an echinoderm microtubule-associated protein-like 4-ALK rearrangement mutation. Further workup found no bone, brain, or abdominal metastases. The final diagnosis was stage IVA adenocarcinoma (Fig. 1A).

She was treated with pemetrexed (500 mg/m^2^) plus platinum-based chemotherapy every 3 weeks, and concurrently crizotinib, 250 mg, orally, twice daily. After 2 cycles her symptoms of chest tightness and dyspnea improved markedly, and chest CT showed that the pleural effusion had resolved, and the lesions had decreased in size. Based on Recist 1.1 criteria, she was considered to have a partial response (Fig. 1B).

After 4 cycles of pemetrexed/platinum-based chemotherapy with concurrent crizotinib she almost reached a complete response (CR) (Fig. 1C). She was continued on maintenance crizotinib alone, and chest CT, brain magnetic resonance imaging (MRI), abdominal ultrasound, and blood tests were performed every 3 months. Her blood CEA remained normal (<6.00 μg/L).

**Figure 2:**
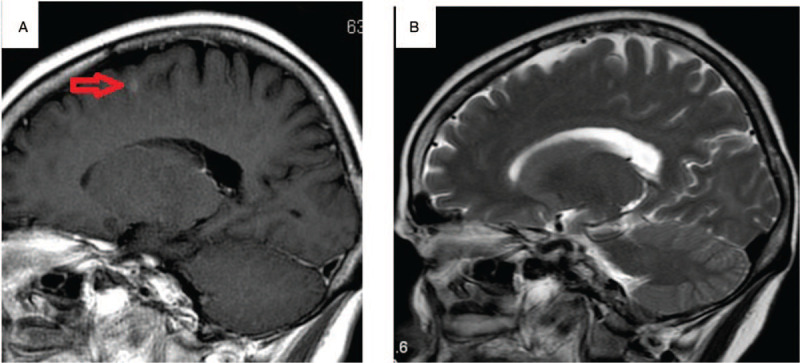
A, Brain metastases in brain MRI. B, Brain metastases have disappeared after ceritinib. MRI = magnetic resonance imaging.

In June, 2020 brain MRI revealed multiple metastases and workup found no metastasis in other organs (Fig. 2A). Her blood CEA level was 50.12 μg/L. Considering cancer progression, but it is difficulty in re-biopsy and NGS for tumor, crizotinib was discontinued and she was begun on ceritinib, 450 mg, orally every day. MRI September, 2020 showed that the brain metastases CR, and her blood CEA level was 5.4 μg/L (Fig. 2B).

**Figure 3:**
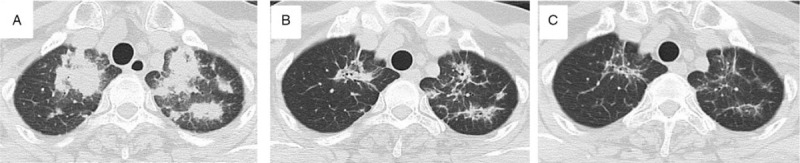
A, Chest CT show multiple patchy lesions appeared in the bilateral upper lungs. B, Treatment 2 weeks later. C, Treatment 1 month later. CT = computer tomography.

On November 20, 2020 she developed an intermittent dry cough and chest tightness, and her body temperature was normal. Her C-reactive protein level was 111 mg/L (normal, ≤ 6 mg/L), her erythrocyte sedimentation rate was 30 mm/h, fungal glucan was normal, and purified protein derivative and tuberculosis-spot were negative too. CT showed multiple patchy lesions in the upper portions of both lungs, and the differential diagnosis was infection or cancer progression (Fig. 3A). Lung function testing showed a decreased forced vital capacity and decreased forced expiratory volume in 1 second.

**Figure 4:**
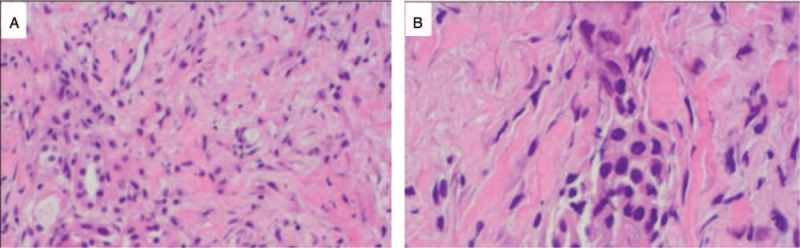
H&E staining showed chronic inflammation. Lymphocyte-predominant and inflammatory cells are present. A, ×200. B, ×400. H-E = hematoxylin-eosin.

She was treated with cefoperazone and levofloxacin for presumed pneumonia. Sputum was obtained via bronchoscopy, and examinations for bacteria, fungi, and tuberculosis were negative. NGS of bronchoalveolar lavage fluid (BAL) was negative. After 1 week of antibiotic treatment there was no change in the appearance of her lungs. A CT-guided needle biopsy of the upper lobe of left lung was performed. Light microscopy examination of a hematoxylin-eosin staining specimen showed numerous reactive inflammatory cells, consistent with lymphocyte-predominant interstitial pneumonitis; no malignant cells were noted (Fig. 4, A and B). The working diagnosis was OP due to ceritinib. Ceritinib was discontinued and she was begun on prednisone, 0.5 mg/kg, orally every day. Two weeks later (Fig. 3B), and 4 weeks later (Fig. 3C), CT showed the lung lesions to be gradually resolving. Tests of lung function were improved. She was continued on prednisone for 2 months and gradually reduced the dose of prednisone every 2 weeks and regularly visited monthly. No related adverse events were occurred in the patient.

**Figure 5:**
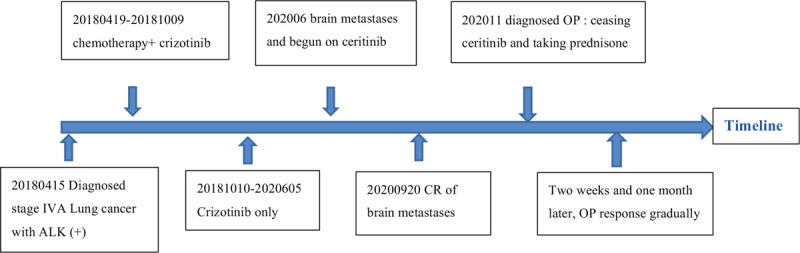
A timeline showed the whole medical procedure of the patient.

A timeline showed the whole medical procedure of the patient (Fig. 5).

## 3 Discussion

As a first-line treatment, crizotinib results in longer PFS as compared with the first-line standard treatment of pemetrexed plus platinum-based chemotherapy in patients with advanced ALK+ NSCLC with ALK (+), with an overall response rate of 75% compared with that of 22% with standard treatment.^[11]^ Crizotinib also has been reported to have a higher overall response rate and prolonged PFS in Chinese patients as compared with chemotherapy (mean PFS 13.3 months vs 5.4 months, respectively).^[12]^ In our patient, chemotherapy combined with concurrent crizotinib as first-line therapy followed by crizotinib alone maintenance therapy resulted in PFS for over 26 months and an almost CR without any adverse events.

Crizotinib, however, has a poor brain penetrance and approximately 41% of patients develop central nervous system metastases. Chemotherapy is not generally useful for brain metastases, while second- and third-generation ALK inhibitors can penetrate the blood–brain barrier and treat brain metastases.^[13]^ Ceritinib has been shown to have significant antitumor activity in patients with ALK+ NSCLC who have received prior crizotinib treatment.^[2]^ Studies have shown that the median duration of response to ceritinib ranges from 7 to 12 months, and when used as a first-line therapy the mean PFS ranges from 16 to 25 months.^[13–15]^

Ceritinib is a second-generation ALK inhibitor, and a dose of 450 mg daily administered with food has a more favorable safety profile in both treatment and pretreated patients with ALK+ metastatic NSCLC.^[16]^ Wu et al^[2]^ reported that overall intracranial response rate and intracranial disease control rate (DCR) in patients with brain metastases treated with ceritinib were 39.1% and 82.6%, respectively, and the mean PFS was 5.7 months. Ceritinib in both post-crizotinib, the response rate was 29.55%. In our patient, treatment with ceritinib resulted in an almost CR of the brain metastases after 3 months.

The most common adverse events associated with ceritinib are diarrhea, vomiting, and an increase in alanine aminotransferase level, which are similar to those of other ALK inhibitors. Lung toxicity is a rare but potentially fatal adverse event also associated with ALK inhibitors, with a reported incidence of 1% to 3%.^[17]^ ALK inhibitor-related pneumonitis is classified as nonspecific interstitial pneumonia, OP, hypersensitivity pneumonitis, and acute interstitial pneumonia, and of these OP is rare.^[10]^

OP is an inflammatory process of the bronchioles that can result in destruction of the small airways and surrounding lung tissue.^[18]^ Chest X-ray may show characteristic patchy unilateral or bilateral lesions, or may show unspecific findings. CT is the most sensitive method to assess involvement and the distribution of lung changes. CT findings are characterized by multiple, predominantly bilateral patchy air space opacities disseminated throughout both lungs, and other findings may include interstitial opacity, focal consolidations, or nodules or masses.^[18]^ High-resolution CT is more sensitive for detecting ground glass infiltrates.^[19]^

OP is relatively rare with characteristic clinical, radiological, and pathological findings and clinical features and imaging studies may resemble those of infectious pneumonia. Approximately 50% of cases of OP are idiopathic, and reported causative factors include drugs, radiation therapy, and chronic inflammatory diseases. The clinical course of OP is frequently similar to that of acute or subacute infectious pneumonia. The condition should be suspected when there is no response to treatment with multiple antibiotics, and blood and sputum cultures are negative.

In patients with OP, cytological findings in BAL samples are characteristic but unspecific; however, BAL is useful for excluding other infectious disorders, tuberculosis, or neoplastic disease. A reliable diagnosis should be based on histopathological analysis of a biopsy specimen (transbronchial, needle, or open biopsy). It is critical to accurately diagnose OP because it must be distinguished from disease progression or metastasis. In our patient, examination of a biopsy specimen showed signs of inflammation and fibrotic changes, implying that OP is a phenotypical immune response induced by ceritinib; however, currently, the exact cause and mechanism of organizing pneumonitis due to treatment with ceritinib is not known.

The overall mortality rate of patients with OP is approximately 10%, and patients usually require treatment with steroids and discontinuation of the ALK inhibitor. Most patients respond to treatment with steroids, and chest distress and dyspnea symptom resolve within days or weeks.^[20]^ Prednisone should be started at a dose of 0.5 to 1.0 mg/kg, or a mean dose of 40 mg (range, 20–120 mg) for 6 weeks, then continued at a lower dosage for 6 to 9 months.^[19]^ Some studies showed that approximately 30% of patients relapses upon withdrawal of treatment or takes ceritinib again. OP may persist and the symptoms could not worsen or recurrence in a minority of patients.

The relatively short follow-up period is a limitation of this report; long-term follow-up is needed to fully evaluate the treatment effect. Second, we do not evaluate OP relapse if cease prednisone completely or take ceritinib again or change other ALK inhibitor.

## 4 Conclusion

OP must be differentiated from the infectious pneumonia, metastasis, or cancer progression, the diagnosis is suspected when there is no response to multiple antibiotics, and blood and sputum cultures are negative, biopsy plays a role in making a diagnosis of OP. It is critical to accurately diagnose OP due to complete difference of those treatments and prognosis. OP usually require treatment with steroids and cease ALK inhibitor. The mechanism of OP is still unknown and needs further research.

## Acknowledgments

The authors thank the patient for permitting them to use her data to complete this article.

## Author contributions

All authors have made substantial contributions to the conception of the work. YHW, HGC, KZ, WBW, LXJ and JZ are the treating doctors. JXG make a pathological diagnosis and provided pathological and IHC photos.

All authors approved the final manuscript as submitted and agree to be accountable for all aspects of the work. The authors report no conflicts of interest.

**Conceptualization:** Yonghui Wu, Huiguo Chen, Kai Zhang, Weibin Wu, Xiaojun Li, Jian Zhang.

**Data curation:** Yonghui Wu, Jiexia Guan.

**Investigation:** Yonghui Wu.

**Writing – original draft:** Yonghui Wu, Huiguo Chen, Jiexia Guan.

**Writing – review & editing:** Yonghui Wu, Jian Zhang.
